# Baby Intensive Early Active Treatment (babiEAT): A Pilot Randomised Controlled Trial of Feeding Therapy for Infants with Cerebral Palsy and Oropharyngeal Dysphagia

**DOI:** 10.3390/jcm12072677

**Published:** 2023-04-03

**Authors:** Amanda Khamis, Nadia Badawi, Catherine Morgan, Iona Novak

**Affiliations:** 1 Cerebral Palsy Alliance Research Institute, Discipline of Child & Adolescent Health, Faculty of Medicine & Health, The University of Sydney, 88 Mallett Street, Camperdown, NSW 2050, Australia; 2 The Children’s Hospital at Westmead, The Sydney Children’s Hospitals Network, Sydney, NSW 2145, Australia

**Keywords:** dysphagia, feeding, infants, cerebral palsy, neuroplasticity, motor-learning

## Abstract

Cerebral palsy (CP), results in impairment of muscle function including the face, mouth, and throat, leading to oropharyngeal dysphagia (OPD), which affects 85% of children with CP. OPD increases risk of deficiencies in growth, neurological development, and aspiration pneumonia, a leading cause of death in CP. This pilot randomised controlled trial aimed to (i) assess feasibility and acceptability of a novel neuroplasticity and motor-learning feeding intervention program, Baby Intensive Early Active Treatment (babiEAT), and standard care, and (ii) explore preliminary efficacy of babiEAT on health and caregiver feeding-related quality of life (QoL). A total of 14 infants with both CP and OPD were randomly allocated to 12 weeks of babiEAT or standard care. Results indicate that babiEAT and standard care are equally feasible, and acceptable. Parents in the babiEAT group thought recommendations were significantly more effective than standard care parents, were more likely to recommend the program to a friend and reported higher QoL. babiEAT infants showed significantly greater efficiency in fluid intake, fewer compensatory strategies with cup drinking, consumption of more advanced food textures, and shorter mealtimes without impacting intake, aspiration risk, or weight. This small pilot study shows promise for babiEAT in infants with CP and OPD. Further research is needed to determine strength of its effects.

## 1. Introduction

### 1.1. Background

Cerebral palsy (CP) is the most common developmental physical disability of childhood and is caused by damage to the developing brain. CP results in impaired motor control throughout the body, including those muscles involved in feeding and swallowing in [1,2]. Oropharyngeal dysphagia (OPD)—the medical term used to describe the symptomatic difficulty or discomfort associated with eating, drinking, or swallowing—is present in 85% of children with CP, with prevalence and severity in direct proportion to the severity of CP [3,4,5,6,7,8,9]. Dysphagia may lead to further impairments such as malnourishment, growth faltering, or further disability, and is a leading cause of death in people who have CP [7,8,10]. While tube feeding methods such as nasogastric and gastrostomy tubes bypass some of these risks, they may lead to prolonged or worsening OPD, and reduce participation, inclusion, and quality of life by removing the mealtime experience altogether [11]. Evidence-based feeding interventions are therefore necessary to reduce the primary and secondary effects of OPD in infants with CP. If proven effective, early intervention for swallowing has the potential to improve long-term outcomes and lower the risk of premature death in infants with CP.

Dysphagia intervention can be grouped into two categories: compensation and skill training. Compensation prioritises immediate safety and function by adapting the task or using alternative equipment to make it easier for the individual to swallow without aspiration [12]. In infants, this includes slowing the flow rate of fluids with slower-flow bottle nipples, increasing the thickness of fluids, modifying the texture of foods to softer or pureed consistencies, and tube feeding [13]. Conversely, skill training aims for the individual to acquire new, more advanced skills and may lead to independence [14,15,16,17,18,19]. Skill training can be split into two more distinct categories: indirect and direct training [12,13,20]. Indirect training aims for skill acquisition through increased resistance and strength training without the use of food or fluid as a stimulus. It can include infant non-nutritive sucking on a pacifier, oral motor resistance exercises with silicone tubes, laryngeal adduction exercises such as the Mendelsohn manoeuvre, and manipulation of non-nutritive items in the mouth [13,20]. Direct training, on the other hand, also aims for new skill acquisition but uses food or fluid during training. It is therefore more task-specific and carries a higher likelihood of producing improved skills acquisition by harnessing neuroplasticity [13,20]. Direct training includes incrementally increasing the flow rate of fluids through faster-flow bottle nipples, titrating down the thickness of fluids, increasing resistance of chewable foods, and controlled exposure to more difficult textured foods.

Research evaluating the effectiveness of OPD interventions is limited and has predominantly focused on adults, older children, or premature infants [13,21]. While some OPD intervention research has been completed with infants with CP, the level of evidence is very low and is insufficient to support or refute the efficacy of the techniques [13]. The evidence that does exist for feeding interventions favours strategies that are based on neuroplasticity and motor learning principles [22]. These principles propose that early, intense practice that is challenging and as close to the task as possible produces the best outcomes [23,24,25]. In light of this emergent evidence, we devised a novel feeding intervention program based on neuroplasticity and motor learning principles (Table 1) called Baby Intensive Early Active Treatment (babiEAT), and tested it empirically compared to standard care. What constitutes standard care is variable; however, a recent international dysphagia survey of practice [20] conducted by the authors of this study demonstrated that, overall, standard care does not align with neuroplasticity and motor learning principles.

### 1.2. Objectives

The aims of the study were as follows: (i) assess the feasibility and acceptability of the babiEAT and standard care feeding interventions; (ii) explore the preliminary efficacy of babiEAT versus standard care on OPD, health, and caregiver feeding-related quality of life (QoL) in infants at high risk of CP with OPD. We hypothesised that both babiEAT and standard care were feasible and acceptable to both infants and their caregivers. We also theorise that infants who receive the babiEAT intervention will have better outcomes and their caregivers will report higher QoL than those who receive standard care.

## 2. Materials and Methods

### 2.1. Trial Design

A pilot evaluator-blinded randomised controlled trial of babiEAT versus Standard Care OPD therapy for infants at risk of CP was conducted and registered on the Australian New Zealand Clinical Trials Registry (Registration Number: ACTRN12618000305224). As this is a pilot RCT, we did not propose a powered study but are exploring the feasibility and acceptability of the babiEAT program and standard care for parents. As there is no available data, no power calculation has been done for this pilot study; the study sample size (*n* = 38; 19 per group) was estimated from an a priori calculation based on best available evidence in premature infants (<37 weeks PMA) or older children where RCTs have been published using various outcome measures pertaining to oral feeding [17,28,29,30,31,32,33,34,35].

Ethics approval was obtained from The Sydney Children’s Hospital Network Human Research Ethics Committee (HREC/17/SCHN/432), Site Specific Approval through Cerebral Palsy Alliance Ethics Committee (2018_06_02), and Clinical Trial Research Agreement with The University of Sydney.

### 2.2. Participants

Infants accessing services through Cerebral Palsy Alliance, Cerebral Palsy Alliance Research Institute, The Sydney Children’s Hospital at Westmead speech pathology department, The Grace Centre for Newborn and Intensive Care, or who were listed on the New South Wales Cerebral Palsy Register between 1 July 2018 and 30 April 2022 were considered for participation in this study. Infants were eligible if they were: term equivalent age through 12 months corrected age at the time of enrolment; diagnosed with CP or at risk of CP, and also had OPD as determined by clinical feeding evaluation (CFE), with a minimum of 20% of nutrition consumed orally. Infants were excluded if their OPD was clinically determined to be primarily of cardiac, respiratory, or gastrointestinal origin; if they had a craniofacial anomaly that impacted feeding; if aspiration risk was unable to be mitigated through compensatory strategies; if they resided in areas not accessible to the principal investigator; if they were not willing or able to carry out the intervention dose; or if caregivers did not speak and read English to a level where they were able to provide informed consent.

### 2.3. Recruitment and Randomisation

**Figure 1:**
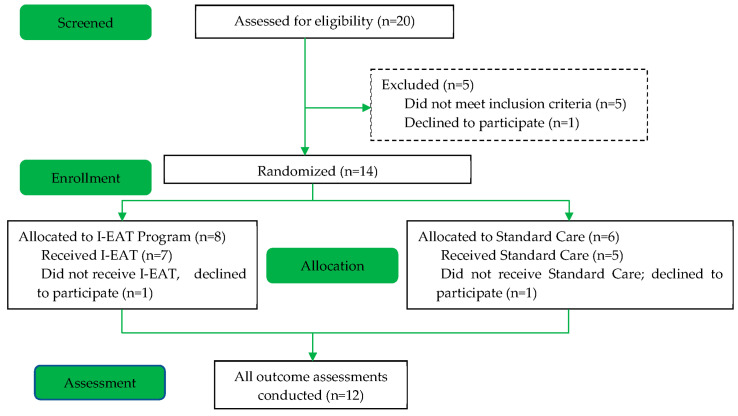
Flow of Participants.

If infants were identified as potentially eligible, the caregivers were provided information about the babiEAT study and a follow-up phone call was provided by the researchers to provide more detail to those who were interested. If the caregiver agreed, an initial assessment was then conducted at the participant’s home to confirm eligibility and obtain consent to participate in the study. Consenting eligible infants underwent baseline assessments and were then randomly allocated to either the babiEAT or standard care group (Figure 1).

Permuted blocked randomisation was conducted by a biostatistician using computer-based sequences, and group allocation was provided in concealed opaque envelopes.

Participants were informed of their group allocation; home visits were scheduled for those randomised to the babiEAT group, and referrals to alternatives services, if required, were given for those in the standard care group.

### 2.4. Interventions

All parents set two Goal Attainment Scaling (GAS) goals, one for fluids and another for solids, collaboratively between caregiver and research speech pathologist at the completion of initial outcome measures. Both groups received 12 weeks of feeding therapy (Table 2) and were re-assessed immediately at the end of their 12-week therapy block.

#### 2.4.1. babiEAT Program

The babiEAT group received twice-weekly home visits for 4 weeks followed by once-weekly home visits for 8 weeks, totalling 12 weeks (Table 2). Intervention was provided in their home setting by a speech pathologist with expertise in feeding difficulties and OPD in infants with CP. babiEAT interventions and home practice were individualised and updated at each session based on the infants’ current skills. The feeding skills of participants in the babiEAT group were reviewed each session to establish whether compensations could be titrated down and whether a more advanced skill was safely tolerated and could therefore be introduced into practice. Recommendations were direct interventions, which followed neuroplasticity and motor learning principles and incorporated food or fluid stimuli. Caregivers of babiEAT infants were instructed to practice these skills 3 times daily for 15 min during snack times, incorporating praise, comments on performance, singing, explorative play with hands, and preferred flavours to make the experience positive and fun for the infant. Those skills or food consistencies that were mastered during snack times were then recommended to be generalised into mealtimes with less extrinsic feedback following a more random and distributed practice model. The novel intervention program and detailed protocol for the babiEAT program is not included in this publication to avoid contaminating standard care intervention and outcomes of future larger-scale RCTs of babiEAT compared to standard care.

#### 2.4.2. Standard Care

Those infants who were randomised to standard care were instructed to continue with their current service for 12 weeks where available (as recommended by their treating speech pathologist), or support was provided to access alternative services (Table 2). Standard care varies widely with no gold-standard guidelines and little research to support clinical decision making. The intervention offered to those infants randomised to standard care was beyond the control of the investigators of this study and could have included compensations and direct or indirect interventions of varying intensities and settings.

### 2.5. Outcomes

The primary outcome of this pilot study was investigating the feasibility and acceptability of both feeding therapy programs. Feasibility and acceptability were determined by caregiver report after 12 weeks of intervention using the Feeding Intervention Preferences Questionnaire (FIPQ), a 9-item 10-point Likert scale questionnaire created by the authors of this study (Supplementary File S1). Secondary outcomes were measured at baseline and immediately following the 12 weeks of intervention. Since part of the premise of the babiEAT program is challenging infants’ feeding skills, there was some risk of possible reduction in feeding efficiency, and an increase in mealtime duration, growth, and aspiration risk.

The secondary measures monitor these potential risks, as well as the improvement in skills and attainment of goals, and included the following:

(i) Oral feeding efficiency: measured by percentage of total volume consumed during first five minutes of the mealtime. It is often recommended that purees and bottle feeding are the focus of intake for young children who have faltering growth as they are perceived to be the most efficient way of consuming nutrition orally. Therefore, there is a risk that feeding efficiency for the babiEAT group may diminish as they are challenged to increase intake of more advanced food and utilise more advanced drinking vessels. Percent volume consumed in the first 5 min is reflective of feeding efficiency [36], with a 30% consumption during this time indicating an efficient feed. Duration of mealtimes was also monitored, as a meal exceeding 30 min is indicative of inefficiency and a red flag for feeding difficulty or OPD [37];
(ii) Feeding and swallowing skills: measured using the Schedule for Oral Motor Assessment (SOMA), recommended International Dysphagia Diet Standardisation Initiative (IDDSI) level, Functional Oral Intake Scale for Infants (FOISi), and the number of compensatory strategies utilised. The SOMA was selected as it is an OPD measurement tool shown to have the strongest validity, reliability, and clinical utility in young children with CP [38]. IDDSI is a universal objective framework for testing and classifying the texture or consistency of food and fluid. Each IDDSI level correlates with the functional skills required to manage that food or fluid; thus, it was employed in this study since change in IDDSI level recommendations is indicative of change in skills [39,40]. The FOISi was used in this study as it is a valid and reliable tool for classifying aspiration risk and OPD severity and evaluating effectiveness of intervention [41]. Compensatory strategies by their nature alter the task and reduce the degree of independence in feeding. They were therefore monitored in this study since a reduction in compensations is clinically associated with an improvement in skill and independent safe feeding.
(iii) Goal Achievement: measured using the GAS, which is valid, reliable, and responsive to clinically significant and functional change in the CP population [42]. T-scores were calculated and used to assess change in GAS goals for both food and fluid goals, as is recommended in literature [42];
(iv) Health: measured by recording presence of known risk factors resulting from OPD in infants with CP, weight recorded as Z-score for age using the World Health Organization growth charts (as is convention for infants under 2-years), plus the number of chest infections and hospitalisations in the three months before and during intervention; and
(v) Feeding-Related Quality of Life: Parents of newborns spend a substantial amount of time feeding their children, and a parent’s perception of success is often intertwined with their ability to nourish their baby. The Feeding Swallowing Impact Scale (FSIS), a valid tool for measuring health-related quality of life, was completed by caregivers to determine the impact and stress that feeding difficulties have on the participants’ caregivers [43]. FSIS scores are categorised into three subtests: those that measure impact of the infants’ feeding difficulties on the caregivers’ ability to carry out daily activities, the caregivers’ worry, and the caregiver’s ability to feed their child. Likert scores for each subtest were combined to create a score for each of the three subtests.

### 2.6. Logbooks

All families were instructed to complete a logbook documenting the number and location of intervention sessions received, time spent carrying out the recommendations from their treating clinician, and any adverse events across the 12 weeks of the study. Regular reminders were made to all families via email to continue to complete this task.

### 2.7. Blinding

Assessments were scored by a speech pathologist with over 15 years of experience in the assessment of infant feeding difficulties and established reliability in the use of all measures. The assessor was masked to group allocation and time-point of the assessment and achieved greater than 90% intra-rater reliability on all measures. All assessments were completed from de-identified videos filmed by the treating speech pathologist. Blinding of participants and treating speech pathologists was not possible due to the nature of the intervention.

### 2.8. Statistical Methods

Statistical analysis was performed using IBM SPSS Statistics for Windows, Version 28.0 (IBM Corp. Released 2021. Armonk, NY, USA: IBM Corp) and reported according to the CONSORT statement (Supplementary File S2). Infant characteristics and baseline measures were compared using independent sample t-tests. As we anticipated that participants would reflect the heterogenous natures of CP and OPD, ANCOVA and logistical regression were used (where baseline scores were entered as covariates) to account for differing baseline severities and between-group differences. ANCOVA was used for outcomes with continuous variables and FIPQ (as scores were combined for each of the three subtests). For outcome measures where there was a possibility that the ceiling may be reached at baseline, scores were dichotomised into either: (i) advanced or maintained maximum/normal, or (ii) regressed or maintained modified/dysfunctional, and binomial logistic regression was conducted to reflect progress and baseline function. As the FIPQ was only conducted at the conclusion of the study, and as data varied in distribution, they were examined using Mann Whitney-U tests, median, and inter-quartile range. Gross motor function severity of CP was not reported, as infants under two years cannot be reliably classified. All data were analysed on an intention-to-treat basis where statistical tests were able to be run with missing data; where this was not possible, they were excluded from analysis.

### 2.9. Impact of COVID-19 Pandemic

This study was impacted by the COVID-19 pandemic. The imposition of restrictions necessitated adaptations to the study protocol. These included conducting some intervention sessions via telehealth during mandated lockdowns and mandated service provision restrictions. The telehealth format included direct instruction, whereby the speech pathologist provided verbal descriptions and physical demonstrations of techniques with dolls and local equipment. All necessary equipment and resources were posted to participants receiving telehealth services including thickener, bottle nipples, bottles, cups, and spoons to allow for continuation of trials with the speech pathologist’s guidance. To mitigate limitations in telehealth audio quality and optimise the ability to detect subtle signs of aspiration risk, lapel microphones were clipped to infants’ clothing, as close to their mouth and neck as possible, in participants at risk of aspiration. In addition to the above, the pandemic lowered rates of referrals and recruitment rates, thus resulting in a lower sample size than originally planned. These issues were attributed to infection-control issues, rather than feasibility or acceptability of the babiEAT program itself.

## 3. Results

### 3.1. Baseline Characteristics

Fourteen infants with a mean age of 9.5 months (SD = 2.03) met eligibility criteria and were randomised (see Figure 1 for a summary of the flow of participants through the study). Although recruitment was open to infants who were exclusive milk-feeders, all participants had commenced intake of puree/solids prior to enrolment. Eight infants were randomised to babiEAT and six to standard care. Two participants, one from each group, withdrew from the intervention after randomisation but prior to intervention commencing, due to changes in the families’ priorities. Post-intervention outcome data for these two participants were therefore not available and could not be included in the analysis. Participant characteristics and baseline outcome measures are summarised in Table 3. Groups were equivalent at baseline with the exception of three factors, and statistical analysis was selected to account for differences in these baseline scores: (i) participants in the standard care group weighed less at baseline (Z-score mean = −0.62, SD = 1.59) than those in the babiEAT group (Z-score mean = −0.09, SD = 0.59) (*p* = 0.03); (ii) participants in the standard care group were less efficient with fluid intake at baseline (*p* = 0.01), with a mean of 32.28% (SD = 14.99) consumed in the first 5 min, compared to 41.27% (SD = 35.61); however, as 30% intake is deemed the cut-off for efficient feeding, the mean of both groups are classified as efficient; (iii) participants in the babiEAT group required more compensation (mean = 9.63, SD = 1.06) than standard care participants (mean = 8.50, SD = 2.35) with cup drinking at baseline (*p* = 0.01).

### 3.2. Intervention

Dysphagia intervention for all participants was delivered by speech pathologists experienced in OPD for infants with CP and results summarised in Table 4.

#### 3.2.1. Location

Participants in the babiEAT group received all sessions in their home during the 12-week period. Only two standard care participants received sessions in their home, one at a rate of 25%, the other 50%, with the remaining sessions conducted in the clinic. Three participants in the babiEAT group received some intervention sessions in their home via video telehealth due to COVID-19 restrictions (median percentage of sessions via telehealth = 0%, IQR = 0–47%). No participants in the standard care group required telehealth sessions.

#### 3.2.2. Intensity

All babiEAT participants received 16 sessions: 8 twice-weekly sessions in the first 4 weeks, followed by 8 weekly sessions in the remaining 8-weeks (100% adherence rate). Standard care ranged from no sessions (*n* = 2) to a maximum of four sessions (median = 2, IQR = 0–4) during the 12-week period. babiEAT caregivers reported a mean of 53.43 h (SD = 0.49) of home practice across the 12-weeks, while standard care caregivers reported a median of 9.9 h (SD = 6.59).

#### 3.2.3. Type of Intervention

All babiEAT participants received individualised interventions and recommendations following neuroplasticity and motor learning principles, combining compensation and direct interventions. Of those standard care participants who received services, 67% received a combination compensation and direct, while 33% received compensations alone.

### 3.3. Feasibility and Acceptability

All caregivers of infants who received intervention completed the FIPQ at the end of the 12 weeks. Eight of the nine questions were scored on a scale of 1–10, with 1 being the poorest result and 10 indicating the highest result, with a total possible score of 80. The median total score of these questions for babiEAT participants was 79 (IQR = 65–80) and median for standard care participants was 77 (IQR = 51–80). Question 3 was also scored on a scale of 1–10; however, 5 was the ideal result indicating that the 12-week program duration was not too long or too short, with both groups reporting a median of 5 (babiEAT IQR = 2–5, Standard Care IQR = 5–5). There was no significant between-group difference on the total score of all nine questions on the FIPQ (*p* = 0.88). Statistically significant between-group differences were found in two questions, Question 2 and Question 4 (Supplementary File S1). Caregivers of children in the babiEAT group were reportedly more likely “to recommend this feeding therapy program to a friend” (median = 10, IQR = 9–10) than those who received standard care (median = 5, IQR = 1–9) (*p* = 0.048). babiEAT interventions were perceived by caregivers to be more “effective” (median = 10, IQR = 10–10) than standard care (median = 8, IQR = 6–10) (*p* = 0.048).

### 3.4. Feeding Efficiency

There was a statistically significant difference in feeding efficiency with fluids in favour of the babiEAT group (*p* = 0.03). The mean volume consumed in the babiEAT group increased when compared to baseline results (baseline mean = 41.27, SD = 35.61; end of treatment mean = 44.00, SD = 37.48), while the mean of the standard care group diminished (baseline mean = 32.28, SD = 14.99; end of treatment mean = 29.80, SD = 20.44). There was no between-group difference in the volume of solids consumed (*p* = 0.68).

### 3.5. Swallowing Skills

#### 3.5.1. Fluids

There was no significant difference in SOMA scores for fluids (bottle *p* = 0.31; trainer cup (straw) *p* = 0.19; cup *p* = 0.09), however, a statistically significant within-group reduction of compensations for cup drinking was noted for babiEAT participants compared to the standard care group by the end of intervention (*p* = 0.02). There was no significant different in IDDSI recommendations for fluids; however, only a very small portion of the sample (*n* = 3) were recommended thickened fluids at baseline and as such the analysis may be underpowered. While there was no statistically significant difference in the achievement of GAS goals for fluids (*p* = 0.053), the mean for participants in the babiEAT group increased two standard deviations from baseline (mean = 58.57, SD = 12.15) and surpassed a T-score of 50, indicating that goals were achieved—a clinically significant outcome. The mean T-score for participants in standard care did not reach 50 (mean = 40, SD = 17.32), indicating goals were not achieved.

#### 3.5.2. Solids

There were no significant differences in the percentage of solids consumed in the first 5 min (feeding efficiency) (*p* = 0.63) or GAS goals for solids (*p* = 0.30) between infants in the babiEAT program and those who received standard care. Similarly to fluid GAS goals, the mean solids T-score for participants in the babiEAT group improved over two standard deviations from baseline and surpassed a T-score of 50 (mean = 55.71, SD = 16.188) representing goal attainment, while the mean for those who received standard care remained under 50 (mean = 46, SD = 13.42), indicating goals were not reached; this highlights a clinically significant between-group difference. Those participants who received babiEAT made significantly more progress in both the SOMA Solids subtest (*p* = 0.047) and the recommended IDDSI level for solids (*p* = 0.02). FOISi ratings demonstrate a significant between-group difference in oral intake (*p* = 0.02), further supporting the progression with solids for participants who received babiEAT. While not statistically significant due to small sample size, the improvement in oral intake was further demonstrated by the fact that two babiEAT participants progressing from feeding tube reliance to no longer requiring their feeding tube; by contrast, one participant in the standard care group developed a need for tube feeding at the end of treatment.

### 3.6. Mealtime Duration

While not statistically significant, the mean duration of mealtimes for the standard care group remained over 30 min (mean = 31, SD = 8.22) after 12 weeks of intervention, while the mean duration for the babiEAT group reduced to less than 30 min (mean = 26.43, SD = 3.78), despite the babiEAT mean being higher prior to the intervention.

### 3.7. Health Outcomes

No statistically significant between-group difference was found in Z-score weight measures (*p* = 0.51), indicating that participants in the babiEAT group are at no greater risk of growth or nutritional deficiencies than those who received standard care. There were no instances of chest infection, hospitalisations, or any adverse events (e.g., choking or death) in the babiEAT group, and one instance of chest infection (RSV) in the standard care group.

### 3.8. Quality of Life

babiEAT parents reported significantly higher QoL in all three FSIS subtests for the than standard care (Impact on Daily Activities *p* = <0.001; Worry *p* = <0.001; Problems Feeding Child *p* = 0.03).

## 4. Discussion

This study assessed the feasibility and acceptability of both babiEAT and standard care feeding interventions and explored their preliminary efficacy on feeding skills, health, and caregiver QoL in infants with or at high risk of CP who have OPD.

### 4.1. Evidence-Based Practice

The best available evidence supports the neuroplasticity and motor learning principles on which the babiEAT program is based, with its intensive nature, task-specific recommendations and service, and challenge of skills with regular uplevelling. The results we observed in the standard care group substantiates our international survey of dysphagia practice [20] and clinical practice guidelines for children 0–2 years with CP [22], which highlighted that standard care for OPD most often consists of low-dosage service emphasising compensatory strategies to simplify the task, which does not allow for timely challenge of skills and as such does not align with evidence.

Service delivery between groups was starkly different, with all participants in the babiEAT group receiving an intensive service in their natural (home) environment. In contrast, two participants in the standard care group did not receive any services during the 12 week study period, and no participants in this group received more than four sessions; this intensity also reflects the results seen in our international survey of dysphagia practice. Only two standard care participants received some services in the home setting (25% and 50% of their total services, respectively), with the remainder occurring in out-patient clinics. Provision of services in the natural environment with a child’s own seating and mealtime equipment, where clinicians can observe the process of typical meal preparation, and natural distractions such as people and noises facilitate successful follow-through of intervention and recommendations between sessions. This may have influenced the vastly different hours of home practice between babiEAT and standard care (mean = 53.43 h, 9.9 h, respectively). Interventions for all participants receiving babiEAT closely followed neuroplasticity and motor learning principles primarily through direct interventions, whereas only 40% of the standard care group received recommendations for direct intervention. While compensatory strategies were recommended to all babiEAT participants, they were also all provided opportunity to safely challenge skills, with the need for compensations being re-evaluated frequently and swiftly titrated down as tolerated. Due to the limited intensity of services for standard care participants, it is unlikely that compensations were able to be regularly and promptly titrated down. This finding corroborates our recent findings that standard care does not closely align with the best practice of intervention following neuroplasticity and motor learning principles [20].

### 4.2. Feasibility and Acceptability

High overall FSIS scores across both groups indicate that both the babiEAT and standard care interventions were feasible and acceptable to infants and their caregivers, confirming the need and value of feeding intervention for infants with CP and OPD. Despite the babiEAT program being more intensive, requiring more time investment from caregivers, with perceived higher risks, caregivers of babiEAT participants were more likely to recommend the intervention to a friend and also found the intervention recommendations more effective, compared to those who received standard care. One caregiver in the babiEAT group commented: “we found this eating program extremely helpful! I truly don’t think that we would have the success with feeding that we currently have if it wasn’t for this program.” Adherence to the babiEAT protocol was high for all families, and all caregivers completed the logbook of sessions, home practice, and hospitalisations. One comment from a babiEAT caregiver stated “although it was a lot of appointments in addition to our regular therapies… it wasn’t too much to handle. All the advice and tips given were actually helpful… so it was no task to implement them.” Within the standard care group, those participants who did not receive services during the 12 weeks of the intervention continued with recommendations and home practice as were recommended to them in their last feeding therapy session and recorded these in the logbook. Although one participant in each group withdrew after randomisation and before intervention commenced, both reported this to be due to a change in circumstances, as other developmental areas took priority over feeding. The recruitment process was clinically feasible to carry out and acceptable to all families, although referrals for recruitment were slow, likely due to the COVID-19 pandemic.

### 4.3. Feeding Skill Development

While there were no significant differences in the SOMA or IDDSI scores between groups for drinking skills, this is likely attributable to the very small sample of participants overall and specifically of those requiring thickener at the start of intervention (*n* = 3). Furthermore, as the risk of aspiration with fluids tends to be greater than with solids, compensations such as thickening fluids were likely to have been implemented and would therefore not demonstrate a functional change in SOMA scores; this is a known limitation of the tool. However, the significant reduction in the number of compensations with cup drinking, plus increased efficiency in fluid intake, are promising markers of improved skills with fluids (without an increased risk of aspiration) with the babiEAT program. This finding was supported in improvements by a mean of over two standard deviations in GAS T-scores and overall attainment of fluid goals by babiEAT participants, with the standard care group only reaching one standard deviation above baseline and remaining below the goal attainment level. This signifies a progression in skill development, independence, and function that is both clinically significant and meaningful to caregivers.

Although feeding efficiency and total intake of solids was not significantly different, a significantly positive outcome in management of solids was shown in SOMA scores, IDDSI level, and FOISi. This demonstrates that volume of solid intake can remain consistent whilst advancing skills with solid foods and that advancing feeding skills is an appropriate goal, even for those infants with faltering growth. While the number of compensations for solids was not significantly different between groups, this is likely due to the substantial advancement in the difficulty of foods being consumed, which are likely to require more compensation than the purees which were common at baseline. This is particularly the case for those whose CP may impact their ability to self-feed due to upper limb involvement. Similar to fluid GAS goals, solids GAS T-scores represented goal attainment with a mean of more than two standard deviations higher than baseline for the babiEAT participants, while standard care participants only achieved an increase of one standard deviation and the mean remained below the goal attainment level.

Although the difference in mealtime duration did not reach statistical significance, it is promising to see that the babiEAT group post-intervention had a mean mealtime duration of 26.43 min (SD = 3.78) compared to the standard care mean which increased from sub-30 min at baseline (mean = 29.17, SD = 6.65) to over 30 min after the 12 weeks of intervention (mean = 31.00, SD = 8.22). This is clinically significant, as a mealtime duration of over 30 min is a clinical red flag indicator for feeding difficulties [37,44].

### 4.4. Health

Despite the progression of babiEAT participants towards more challenging foods and modalities of drinking and the perceived associated health risks, the absence of a between-group difference in weight Z-scores, plus the progression of all feeding tube-dependent babiEAT participants (*n* = 2) to fully oral feeders, suggests the risks related to feeding efficiency and oral intake are quite small. Another important health finding of this study was that there were no incidences of chest infection or hospitalisation in the babiEAT group, although there was one occurrence of chest infection in the standard care group.

### 4.5. Quality of Life

Despite the anticipated increased load on caregivers in the babiEAT group from additional appointments and home practice, the difference in FSIS scores for all three subtests indicates that the impact of the infants’ feeding difficulties on caregivers was significantly less after the babiEAT program than a course of standard care. This result may reflect reprieve in a number of areas including (i) receiving additional guidance from a speech pathologist; (ii) a decline in associated risks such as aspiration and choking; (iii) a reduction in OPD and improvement in functional skills; (iv) a reduction in required compensations that can be mentally taxing and time consuming; (v) an overall simplification of the mealtime experience thus increasing the capacity for others to assist in the mealtime process and share the load; plus (vi) a reduction in mealtime duration leaving more time for other valuable daily activities, leisure tasks, and bonding with their child.

### 4.6. Limitations

The small sample size of this study is likely to have limited the levels of significance below what might have been established in a larger sample size, that is, the study is under-powered for efficacy. As participants were under 12 months at the time of enrolment, they were too young to establish their gross motor function classification system (GMFCS) level (minimum age is 2 years), which is prognostic of severity of CP [45]. It is likely that those with a higher level of severity of CP will have a higher severity of OPD, and thus would be less likely to have made progress during this study. All participants had received some feeding intervention prior to enrolling in this study; the level of intervention is unknown; however, it is possible that progress had plateaued for some.

There is only one validated, published clinical feeding or OPD assessment tool that can be used across ages from birth to 18 months of age—the SOMA—which was utilised in this study. There are known limitations with this tool, including its ability to detect aspiration risk and responsiveness to change over time [46,47]. We attempted to compensate for its limitations by also monitoring IDDSI levels, FOISi score, number of compensatory strategies, and GAS goals; however, results may be impacted by the lack of one overarching tool to monitor overall skill attainment and aspiration risk. It was not possible to conduct the gold standard VFSS due to unwarranted exposure to radiation; as such, a more objective measure of reduction in aspiration risk was not possible.

Participants were all from one urban location with access to services within their metropolitan city. It is possible that our results would not be replicable in rural or remote communities with less access to services. However, three of the babiEAT participants received video-telehealth services for part of their intervention and continued to make comparable gains in their skills. This is an encouraging finding as it supports the possibility of translating the babiEAT program to one that can be effectively delivered online, making it more accessible to rural, remote, or potentially to low- and middle-income countries.

### 4.7. Future Directions

As this was a pilot study with a small sample size, our outcomes should be interpreted as exploratory until confirmed by a larger-scale randomised controlled trial. There is opportunity for pursuing this in collaboration with existing intensive infant feeding clinic services both in Australia and internationally.

## 5. Conclusions

This pilot RCT demonstrated that 12 weeks of feeding and OPD intervention for infants under 12 months with both CP and OPD is feasible and acceptable to infants and caregivers. Importantly, the babiEAT program was safe to deliver, and infants made progress from this approach. This work highlighted the positive impacts of a neuroplasticity and motor learning principle-based program, babiEAT, on oral feeding skills with both food and fluids: more efficient mealtimes, with higher QoL for caregivers, and no negative impacts on health or growth as a result of challenging infants’ skills. Adaptation of the babiEAT program to video-telehealth provision also appears promising.

## Figures and Tables

**Table 1:** Application of Neuroplasticity and Motor Learning Principles to Feeding Interventions.

Principles of Neuroplasticity	Principles of Motor Learning	Application to Feeding Interventions
Use it or lose it [23,24]		Feeding skills may plateau or diminish if infant receives no or limited opportunity to utilize oropharyngeal skills for feeding and/or oral exploration.
Timing [23,24]		Begin intervention as early as possible i.e., with infants under 12 months.
Maximize opportunity for intensive practice [23,24,25]		Use it and improve it. Repetition is essential for skill acquisition. Feeding skills may improve if opportunity is given to safely practice challenging skills. Intense practice and support during early learning phase and tapered off as skill is mastered.
Specificity [23,24,25]		Practice during task as close to meal as possible i.e., interventions involving food/fluid and swallowing (while closely monitoring safety)
Simplification [25]		If tasks are too difficult, infants are at risk of developing maladaptive motor patterns. Compensatory strategies can assist in motor learning if they are used to simplify the task in early stages and are titrated down as soon as is safe to do so, to work towards performing the task unaided. Strategies include modifying texture or sensory features of food or fluid, using different drinking vessels, or slowing the rate of the feed.
Attention and motivation [23,24]		Salient and interesting tasks can be achieved during snacks and at meals using preferred flavours and praise from caregivers.
	Extrinsic feedback [23,24]	Knowledge of performance, remaining time or mouthfuls (with simple visuals/prompts where appropriate) and praise provided frequently during early learning and intermittently as skill is mastered
	Implicit learning [23,24]	Natural positive reinforcement and feedback provided through enjoyment of food flavours and reduction in deleterious responses e.g., gagging, coughing, choking
	Blocked practice [23,24]	Practicing desired skill with one food or drink
	Random practice [23,24]	Alternate fluid, chewable and non-chewable textures with every mouthful, once mastered in isolation
	Distributed practice [23,24]	Practice skill during many (short) sessions over a longer period (e.g., snacks throughout the day)
	Complexity [23,24]	Continual reassessment, upgrade goals, and reducing support to ensure task is as difficult as possible while maintaining safety
	Transfer of learning [26,27]	Once skill is mastered with familiar foods in familiar environments, skills can be practiced with novel foods and environments to encourage generalisation of skills.

**Table 2:** Comparison of Interventions.

	Intensive Early Active Treatment (babiEAT) Program	Standard Care
HOW MUCH	60-min sessions twice weekly for 4 weeks followed by 60 min sessions once weekly for 8 weeks	As per service protocol, which varies but is typically weekly-to-monthly
HOW LONG	12 weeks	12 weeks
WHERE	Participant’s home	Clinical setting or home, as per service protocol
WHO	Individual session, caregiver actively involved	Individual or group or consultation, as per service protocol
WHAT	Individualised skill-building recommendations and home program based on feeding pathophysiology, neuroplasticity, and motor learning principles: ○ intensive, practice 15 mins 3 times daily; ○ early, from 37 weeks PMA; ○ motivating; ○ positive reinforcement; ○ during functional feeding tasks; ○ desired skill is repeated several times each day; ○ simplification (compensatory strategies) as needed and titrated down as tolerated; ○ blocked followed by random practice; ○ skill is generalised to meals as soon as mastered. Skills re-evaluated at each session and recommendations updated to ensure skills are continually challenged (while maintaining safety); Home-based program with a focus on up-skilling caregivers on identifying and responding to their child’s feeding readiness and stop cues and aspiration risk, and coaching caregivers to implement skill-building interventions for 15 min 3 times daily.	Standard practice is likely to be variable. Intervention will be determined by the clinician providing the intervention and as per service protocol.
HOW WELL	Caregivers were instructed to complete a logbook of adverse events and time spent on home program.	Caregivers were instructed to keep a logbook of adverse events, number of sessions, recommendations, and time spent on home program.

PMA: Post Menstrual Age.

**Table 3:** Baseline characteristics of participants.

Characteristic	babiEAT (*n* = 8)	Standard Care (*n* = 6)	*p* Value
Corrected age at baseline, mean (SD), months	9.63 (2.33)	9.33 (1.75)	0.46
Sex: M/F	4/4	2/4	
Weight Z-Scores	−0.09 (0.59)	−0.62 (1.59)	0.03
Percentage of volume consumed in first 5 min-fluids	41.27 (35.61)	32.28 (14.99)	0.01
Percentage of volume consumed in first 5 min-solids	68.75 (25.99)	44.5 (22.26)	0.38
Mealtime duration	38.13 (6.51)	29.17 (6.65)	0.54
SOMA Bottle: Normal/Dysfunctional	6/2	3/3	
SOMA Trainer Cup: Normal/Dysfunctional	2/6	1/5	
SOMA Cup: Normal/Dysfunctional	1/7	2/4	
SOMA Puree: Normal/Dysfunctional	3/5	3/3	
SOMA Semi-Solids: Normal/Dysfunctional	2/6	3/3	
SOMA Solids: Normal/Dysfunctional	1/7	0/6	
SOMA Cracker: Normal/Dysfunctional	2/6	1/5	
IDDSI Level 0-Thin Fluids	*n* = 5	*n* = 3	
IDDSI Level 1-Slightly Thick Fluids	*n* = 3	*n* = 3	
IDDSI Level 2-Moderately Thick Fluids	*n* = 0	*n* = 0	
IDDSI Level 3-Extremely Thick Fluids	*n* = 0	*n* = 0	
IDDSI Level 4–Puree	*n* = 3	*n* = 2	
IDDSI Level 5-Minced & Moist	*n* = 4	*n* = 3	
IDDSI Level 6–Soft	*n* = 0	*n* = 1	
IDDSI Level 7–Regular	*n* = 1	*n* = 0	
FOISi Level 1	*n* = 0	*n* = 0	
FOISi Level 2	*n* = 0	*n* = 0	
FOISi Level 3	*n* = 2	*n* = 0	
FOISi Level 4	*n* = 5	*n* = 5	
FOISi Level 5	*n* = 1	*n* = 1	
Number of Compensations-Bottle, mean (SD)	2.25 (1.83)	3.50 (2.35)	1.00
Number of Compensations–Straw, mean (SD)	7.88 (3.36)	8.33 (2.66)	0.61
Number of Compensations-Cup, mean (SD)	9.63 (1.06)	8.50 (2.35)	0.01
Number of Compensations-Puree, mean (SD)	2.25 (2.05)	4.67 (2.50)	0.78
Number of Compensations-Minced and Moist, mean (SD)	6.00 (3.63)	5.83 (2.40)	0.14
Number of Compensations-Soft/Regular, mean (SD)	8.00 (3.74)	8.83 (2.86)	0.33
Number of Compensations-Transitional, mean (SD)	4.50 (3.74)	5.83 (2.48)	0.33
GAS t-score-Fluids, mean (SD)	25.46 (0)	25.46 (0)	-
GAS t-score-Solids, mean (SD)	25.46 (0)	25.46 (0)	-
Chest Infections in Past 3 months	0.63 (0.52)	0.33 (0.52)	0.77
Hospitalisations in Past 3 months	0.63 (0.52)	0.33 (0.52)	0.77
FSIS Impact on Daily Activities	15.13 (5.79)	14.33 (6.41)	0.77
FSIS Worry	23.25 (6.18)	23.17 (5.98)	0.85
FSIS Problems Feeding Child	12.63 (4.24)	16.17 (5.67)	0.92

SOMA: Schedule for Oral Motor Assessment; IDDSI: International Dysphagia Diet Standardisation Initiative; FOISi: Functional Oral Intake Scale for Infants; GAS: Goal Attainment Scaling; FSIS: Feeding Swallowing Impact Scale.

**Table 4:** Outcome Measures.

Outcome Measure	babiEAT (*n* = 7)	Standard Care (*n* = 5)	*p* Value
Number of therapy sessions received, median, (IQR)	16 (16–16)	2 (0–4)	<0.001 *
Hours spent on home program, mean (SD)	53.43 (0.49)	9.9 (6.59)	<0.001 *
FIPQ Q1, median, (IQR)	10 (8–10)	5 (1–10)	0.07
FIPQ Q2, median, (IQR)	10 (9–10)	5 (1–9)	0.048 *
FIPQ Q3, median, (IQR)	5 (2–8)	5 (5–5)	0.27
FIPQ Q4, median, (IQR)	10 (10–10)	8 (6–10)	0.048 *
FIPQ Q5, median, (IQR)	9 (7–10)	9 (6–10)	0.64
FIPQ Q6, median, (IQR)	10 (9–10)	8 (5–10)	0.27
FIPQ Q7, median, (IQR)	10 (10–10)	8 (5–10)	0.15
FIPQ Q8, median, (IQR)	10 (9–10)	9 (6–10)	0.28
FIPQ Q9, median, (IQR)	10 (8–10)	9 (7–10)	0.64
Percentage of volume consumed in first 5 min-fluids	44.00 (37.48)	29.80 (20.44)	0.03 *
Percentage of volume consumed in first 5 min-solids	28.29 (33.97)	35.80 (38.86)	0.63
Mealtime duration (mins)	26.43 (3.78)	31.00 (8.22)	0.53
SOMA Bottle: Advanced or Maintained Normal/Regressed or Maintained Dysfunctional	6/1	3/2	0.31
SOMA Trainer Cup (straw): Advanced or Maintained Normal/Regressed or Maintained Dysfunctional	4/3	1/4	0.19
SOMA Cup: Advanced or Maintained Normal/Regressed or Maintained Dysfunctional	6/1	2/3	0.09
SOMA Puree: Advanced or Maintained Normal/Regressed or Maintained Dysfunctional	6/1	2/3	0.09
SOMA Semi-Solids (Minced and Moist or Soft): Advanced or Maintained Normal/Regressed or Maintained Dysfunctional	4/3	3/2	0.92
SOMA Solids (Regular): Advanced or Maintained Normal/Regressed or Maintained Dysfunctional	3/4	0/5	0.047 *
SOMA Cracker (Transitional): Advanced or Maintained Normal/Regressed or Maintained Dysfunctional	3/4	1/4	0.40
IDDSI Fluids: Advanced or Maintained Maximum/Regressed or Maintained Modified	6/1	5/0	0.29
IDDSI Solids: Advanced or Maintained Maximum/Regressed or Maintained Modified	6/1	1/4	0.02 *
FOIS: Advanced or Maintained Maximum/Regressed or Maintained Dysfunctional	6/1	1/4	0.02 *
Number of Compensations-Bottle, mean (SD)	0.71 (1.11)	2.60 (3.44)	0.28
Number of Compensations-Straw, mean (SD)	5.00 (3.61)	7.20 (3.90)	0.36
Number of Compensations-Cup, mean (SD)	4.14 (2.67)	6.80 (3.27)	0.02 *
Number of Compensations-Puree, mean (SD)	0.71 (1.89)	1.80 (2.68)	0.98
Number of Compensations-Minced and Moist or Soft, mean (SD)	3.00 (3.56)	6.20 (3.56)	0.11
Number of Compensations-Regular, mean (SD)	4.14 (4.38)	8.00 (2.74)	0.19
Number of Compensations-Transitional, mean (SD)	2.57 (3.74)	4.80 (3.11)	0.42
GAS T-score-Fluids, mean (SD)	58.57 (12.15)	40.00 (17.32)	0.05
GAS T-score-Solids, mean (SD)	55.71 (16.18)	46 (13.42)	0.30
Weight Z-Scores (WHO)	35.99 (34.82)	32.19 (34.40)	0.51
Chest Infections in Past 3 months	0.00 (0.00)	0.020 (0.45)	0.14
Hospitalisations in Past 3 months	0.00 (0.00)	0.020 (0.45)	0.14
FSIS Impact on Daily Activities	10.86 (3.24)	15.00 (4.00)	<0.001 *
FSIS Worry	16.43 (5.32)	24.40 (3.29)	<0.001 *
FSIS Problems Feeding Child	9.29 (2.93)	17.20 (4.01)	0.03 *

* = indicates a statistically significant between-group difference. FIPQ: Feeding Intervention Preference Questionnaire; SOMA: Schedule for Oral Motor Assessment; IDDSI: International Dysphagia Diet Standardisation Initiative; FOISi: Functional Oral Intake Scale for Infants; GAS: Goal Attainment Scaling; FSIS: Feeding Swallowing Impact Scale.

## Data Availability

The data presented in this study are available on request from the corresponding author. The data are not publicly available due to privacy.

## Supplementary Materials

The following supporting information can be downloaded at: https://www.mdpi.com/article/10.3390/jcm12072677/s1. File S1. Feeding Intervention Preference Questionnaire. File S2. CONSORT 2010 checklist of information to include when reporting a randomised trial.

## References

[1] Rosenbaum P., Paneth N., Leviton A., Goldstein M., Bax M., Damiano D., Dan B., Jacobsson B. (2007). A report: The definition and classification of cerebral palsy April 2006. Dev. Med. Child Neurol. Suppl..

[2] McIntyre S., Goldsmith S., Webb A., Ehlinger V., Hollung S.J., McConnell K., Arnaud C., Smithers-Sheedy H., Oskoui M., Khandaker G. (2022). Global prevalence of cerebral palsy: A systematic analysis. Dev. Med. Child Neurol..

[3] Benfer K.A., Weir K.A., Bell K.L., Ware R.S., Davies P.S., Boyd R.N. (2013). Oropharyngeal dysphagia and gross motor skills in children with cerebral palsy. Pediatrics.

[4] Calis E.A., Veugelers R., Sheppard J.J., Tibboel D., Evenhuis H.M., Penning C. (2008). Dysphagia in children with severe generalized cerebral palsy and intellectual disability. Dev. Med. Child Neurol..

[5] Erkin G., Culha C., Ozel S., Kirbiyik E.G. (2010). Feeding and gastrointestinal problems in children with cerebral palsy. Int. J. Rehabil. Res..

[6] Sullivan P., Lambert B., Rose M., Ford-Adams M., Johnson A., Griffiths P. (2000). Prevalence and severity of feeding and nutritional problems in children with neurological impairment: Oxford Feeding Study. Dev. Med. Child Neurol..

[7] Reid S.M., Carlin J.B., Reddihough D.S. (2012). Survival of individuals with cerebral palsy born in Victoria, Australia, between 1970 and 2004. Dev. Med. Child Neurol..

[8] Strauss D., Cable W., Shavelle R. (1999). Causes of excess mortality in cerebral palsy. Dev. Med. Child Neurol..

[9] Reilly S., Skuse D., Poblete X. (1996). Prevalence of feeding problems and oral motor dysfunction in children with cerebral palsy: A community survey. J. Pediatr..

[10] Cerebral Palsy Alliance Australian Cerebral Palsy Register Report 2018. https://cpregister.com/wp-content/uploads/2019/02/Report-of-the-Australian-Cerebral-Palsy-Register-Birth-Years-1995-2012.pdf.

[11] Goday P.S., Huh S.Y., Silverman A., Lukens C.T., Dodrill P., Cohen S.S., Delaney A.L., Feuling M.B., Noel R.J., Gisel E. (2019). Pediatric Feeding Disorder: Consensus Definition and Conceptual Framework. J. Pediatr. Gastroenterol. Nutr..

[12] Logemann J.A. (1991). Approaches to management of disordered swallowing. Baillière’s Clin. Gastroenterol..

[13] Khamis A., Novak I., Morgan C., Tzannes G., Pettigrew J., Cowell J., Badawi N. (2020). Motor Learning Feeding Interventions for Infants at Risk of Cerebral Palsy: A Systematic Review. Dysphagia.

[14] Arvedson J., Clark H., Lazarus C., Schooling T., Frymark T. (2010). Evidence-based systematic review: Effects of oral motor interventions on feeding and swallowing in preterm infants. Am. J. Speech-Lang. Pathol..

[15] Arvedson J., Clark H., Lazarus C., Schooling T., Frymark T. (2010). The effects of oral-motor exercises on swallowing in children: An evidence-based systematic review. Dev. Med. Child Neurol..

[16] Greene Z., O’Donnell C.P., Walshe M. (2016). Oral stimulation for promoting oral feeding in preterm infants. Cochrane Database Syst. Rev..

[17] Boiron M., Nobrega L.D., Roux S., Henrot A., Saliba E. (2007). Effects of oral stimulation and oral support on non-nutritive sucking and feeding performance in preterm infants. Dev. Med. Child Neurol..

[18] Fucile S., Gisel E., Lau C. (2002). Oral stimulation accelerates the transition from tube to oral feeding in preterm infants. J. Pediatr..

[19] Fucile S., McFarland D.H., Gisel E.G., Lau C. (2012). Oral and nonoral sensorimotor interventions facilitate suck-swallow-respiration functions and their coordination in preterm infants. Early Hum. Dev..

[20] Khamis A., Badawi N., Morgan C., Galea C., Novak I. (2023). International Survey of Dysphagia Practice; Practice and Evidence Don’t Align.

[21] Zimmerman E., Carnaby G., Lazarus C.L., Malandraki G.A. (2020). Motor Learning, Neuroplasticity, and Strength and Skill Training: Moving From Compensation to Retraining in Behavioral Management of Dysphagia. Am. J. Speech Lang. Pathol..

[22] Morgan C., Fetters L., Adde L., Badawi N., Bancale A., Boyd R.N., Chorna O., Cioni G., Damiano D.L., Darrah J. (2021). Early Intervention for Children Aged 0 to 2 Years with or at High Risk of Cerebral Palsy: International Clinical Practice Guideline Based on Systematic Reviews. JAMA Pediatr..

[23] Robbins J., Butler S.G., Daniels S.K., Gross R.D., Langmore S., Lazarus C.L., Martin-Harris B., McCabe D.J., Musson N.D., Rosenbek J.C. (2008). Swallowing and dysphagia rehabilitation: Translating principles of neural plasticity into clinically oriented evidence. J. Speech Lang. Hear. Res..

[24] Kleim J.A., Jones T.A. (2008). Principles of experience-dependent neural plasticity: Implications for rehabilitation after brain damage. J. Speech Lang. Hear. Res..

[25] Sheppard J.J. (2008). Using motor learning approaches for treating swallowing and feeding disorders: A review. Lang. Speech Hear. Serv. Sch..

[26] Gillman A., Winkler R., Taylor N.F. (2017). Implementing the Free Water Protocol does not Result in Aspiration Pneumonia in Carefully Selected Patients with Dysphagia: A Systematic Review. Dysphagia.

[27] Gosa M., Schooling T., Coleman J. (2011). Thickened Liquids as a Treatment for Children With Dysphagia and Associated Adverse Effects. ICAN Infant Child Adolesc. Nutr..

[28] Lau C., Fucile S., Gisel E.G. (2012). Impact of nonnutritive oral motor stimulation and infant massage therapy on oral feeding skills of preterm infants. J. Neonatal-Perinat. Med..

[29] Gisel E.G. (1996). Effect of oral sensorimotor treatment on measures of growth and efficiency of eating in the moderately eating-impaired child with cerebral palsy. Dysphagia.

[30] Ottenbacher K., Scoggins A., Wayland J. (1981). The effectiveness of a program of oral sensory-motor therapy with the severely and profoundly developmentally disabled. Occup. Ther. J. Res..

[31] Sjögreen L., Tulinius M., Kiliaridis S., Lohmander A. (2010). The effect of lip strengthening exercises in children and adolescents with myotonic dystrophy type 1. Int. J. Pediatr. Otorhinolaryngol..

[32] Serel Arslan S., Demir N., Karaduman A.A. (2017). Effect of a new treatment protocol called Functional Chewing Training on chewing function in children with cerebral palsy: A double-blind randomised controlled trial. J. Oral Rehabil..

[33] Bernbaum J.C., Pereira G.R., Watkins J.B., Peckham G.J. (1983). Nonnutritive sucking during gavage feeding enhances growth and maturation in premature infants. Pediatrics.

[34] Sehgal S., Prakash O., Gupta A., Mohan M., Anand N. (1990). Evaluation of beneficial effects of nonnutritive sucking in preterm infants. Indian Pediatr..

[35] White-Traut R.C., Nelson M.N., Silvestri J.M., Vasan U., Littau S., Meleedy-Rey P., Gu G., Patel M. (2002). Effect of auditory, tactile, visual, and vestibular intervention on length of stay, alertness, and feeding progression in preterm infants. Dev. Med. Child Neurol..

[36] Griffith T., Rankin K., White-Traut R. (2017). The Relationship Between Behavioral States and Oral Feeding Efficiency in Preterm Infants. Adv. Neonatal Care.

[37] Weir K., McMahon S., Barry L., Masters I.B., Chang A.B. (2009). Clinical signs and symptoms of oropharyngeal aspiration and dysphagia in children. Eur. Respir. J..

[38] Benfer K.A., Weir K.A., Boyd R.N. (2012). Clinimetrics of measures of oropharyngeal dysphagia for preschool children with cerebral palsy and neurodevelopmental disabilities: A systematic review. Dev. Med. Child Neurol..

[39] Cichero J.A.Y. (2020). Evaluating chewing function: Expanding the dysphagia field using food oral processing and the IDDSI framework. J. Texture Stud..

[40] Su M., Zheng G., Chen Y., Xie H., Han W., Yang Q., Sun J., Lv Z., Chen J. (2018). Clinical applications of IDDSI framework for texture recommendation for dysphagia patients. J. Texture Stud..

[41] Yi Y.G., Shin H.I. (2019). Psychometrics of the Functional Oral Intake Scale for Infants. Front. Pediatr..

[42] Cusick A., McIntyre S., Novak I., Lannin N., Lowe K. (2006). A comparison of goal attainment scaling and the Canadian Occupational Performance Measure for paediatric rehabilitation research. Pediatr. Rehabil..

[43] Lefton-Greif M.A., Okelo S.O., Wright J.M., Collaco J.M., McGrath-Morrow S.A., Eakin M.N. (2014). Impact of children’s feeding/swallowing problems: Validation of a new caregiver instrument. Dysphagia.

[44] Arvedson J.C. (2013). Feeding children with cerebral palsy and swallowing difficulties. Eur. J. Clin. Nutr..

[45] Gorter J.W., Ketelaar M., Rosenbaum P., Helders P.J., Palisano R. (2009). Use of the GMFCS in infants with CP: The need for reclassification at age 2 years or older. Dev. Med. Child Neurol..

[46] Ko M.J., Kang M.J., Ko K.J., Ki Y.O., Chang H.J., Kwon J.Y. (2011). Clinical Usefulness of Schedule for Oral-Motor Assessment (SOMA) in Children with Dysphagia. Ann. Rehabil. Med..

[47] Speyer R., Cordier R., Parsons L., Denman D., Kim J.H. (2018). Psychometric Characteristics of Non-instrumental Swallowing and Feeding Assessments in Pediatrics: A Systematic Review Using COSMIN. Dysphagia.

