# Supplementary material for: Baby Intensive Early Active Treatment (babiEAT): A Pilot Randomised Controlled Trial of Feeding Therapy for Infants with Cerebral Palsy and Oropharyngeal Dysphagia
Source: J Clin Med. 2023 Apr 3;12(7):2677. doi: 10.3390/jcm12072677 (PMC10095351; doi:10.3390/jcm12072677)
Supplement: Supplementary file 1 [file jcm-12-02677-s001.zip › S1 Feeding Intervention Preference Questionnaire.pdf]

Feeding Intervention Preference Questionnaire

To be completed by parents/carers.

We are interested to know what you thought of the I-EAT/Standard Treatment Program that you and your child received. Please fill out this questionnaire truthfully, your thoughts will be used to shape our therapy program in the future.

If you have any additional comments, please add them below:

Feeding Intervention Preference Questionnaire

1. How likely would you be to participate in this feeding therapy program again?

1	2	3	4	5	6	7	8	9	10
Not likely									Very likely

2. How likely would you be to recommend this feeding therapy program to a friend?

1	2	3	4	5	6	7	8	9	10
Not likely									Very likely

3. What did you think of the length of the feeding therapy program (12-weeks)?

1	2	3	4	5	6	7	8	9	10
Too short									Too long

4. What did you think of the feeding therapy recommendations?

1	2	3	4	5	6	7	8	9	10
Not effective									Very effective

5. How easy was it to fit the recommendations into your life?

1	2	3	4	5	6	7	8	9	10
Very difficult									Very easy

6. How customised were the recommendations to your child's current feeding skills?

1	2	3	4	5	6	7	8	9	10
Not customised									Perfectly customised

7. How supported did you feel during the feeding therapy program?

1	2	3	4	5	6	7	8	9	10
Not supported									Very supported

8. How convenient were the therapy sessions?

1	2	3	4	5	6	7	8	9	10
Not convenient									Very convenient

9. How likely are you to keep using the techniques you learned?

1	2	3	4	5	6	7	8	9	10
Definitely would not									Definitely will
